# Power Spectral Density Analysis of Solid-State Nanopore Signals: Application to Stability Estimation

**DOI:** 10.1021/acsomega.6c00036

**Published:** 2026-04-14

**Authors:** Pratima Upretee, Eric Beamish, Wouter Botermans, Matteo Pero Cartiglia, Nilesh Madhu

**Affiliations:** † IDLab, Department of Electronics and Information Systems, Ghent University - imec, Technologiepark Zwijnaarde 122, Ghent 9052, Belgium; ‡ imec, Kapeldreef 75, Leuven 3001, Belgium

## Abstract

Reliable solid-state nanopore sensing requires stable, low-noise ionic current baselines. Existing stability evaluation methods often rely on subjective visual inspection. Here, we propose a robust quantitative framework that assesses nanopore wettedness and stability indicators through power spectral density (PSD) analysis of the noise floor in the measured ionic current and uses the resulting features to predict wettedness. A systematic comparison of multiple PSD fitting models and weighting strategies identified a five-component, five-parameter (5C5P) model coupled with high-frequency, low-PSD (HFLS) weighting as the most accurate and consistent approach for characterizing noise in the measured ionic current. Utilizing the noise coefficients from this optimized noise PSD fit, we applied logistic regression to predict nanopore wettedness. Among these coefficients, the 1/*f* noise coefficient and the white-noise coefficient, together with a compact measure of overall low-frequency noise and the applied voltage, formed a compact and interpretable feature set for distinguishing wetted and unwetted pores. A logistic regression classifier trained on these features achieved high performance across a mixed-voltage dataset (median F1-score = 98%) and generalized effectively to unseen applied voltages and different pore dimensions. Segment-wise evaluation on one-second windows that mimic real-time operation demonstrated that the classifier reliably distinguished wetted and unwetted pores across all applied voltages. This physics-informed, data-driven framework enables automated wettedness prediction and stability assessment, indicating a pathway toward reliable real-time quality control for multiplexed, high-throughput solid-state nanopore sensing platforms.

## Graphical abstract

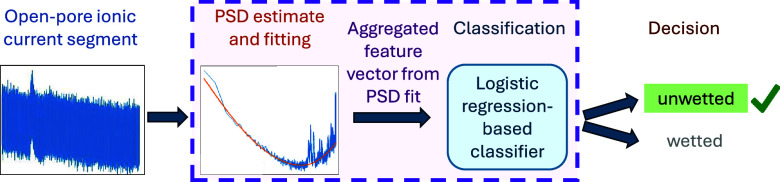

## 1 Introduction

Solid-state nanopores (SSNs) have emerged as promising label-free single-molecule sensors.^1^ Their applications include DNA, RNA, and protein sequencing, single biomolecule identification, and DNA storage.^2–4^ SSNs consist of nanoscale apertures typically fabricated in thin membranes such as silicon nitride, silicon oxide, or graphene.^5^ The fundamental operating principle of SSNs involves detecting ionic current fluctuations driven by an applied voltage across the nanopore, wherein transient reductions in the ionic current reflect the translocation of individual molecules.

Accurate characterization of molecules during nanopore-based translocation experiments requires prior confirmation of nanopore stability. Electrical characterization, involving assessments of resistance, capacitance, noise levels, and baseline stability, is routinely performed to ensure nanopore readiness.^6,7^ Nanopore stability is influenced by multiple factors, notably surface properties, such as wettability. Adequate wetting enables continuous ionic pathways, whereas incomplete wetting can produce nanobubbles, increasing pore resistance and low-frequency (1/*f*) noise,^8,9^ impairing the utility of nanopores for single-molecule detection.^6^ Additional instabilities such as baseline drift and random telegraph noise further degrade event detection accuracy.^10,11^

Although nanopore wettedness alone does not fully define stability, it remains an important indicator of nanopore quality and experimental readiness. Thus, accurate quantification of pore wettedness is essential for achieving reproducible and stable nanopore translocation measurements.^12^ Accordingly, stability as considered in this study refers specifically to wetting-related behavior rather than all possible instability mechanisms.

Surface conditioning with piranha or air/oxygen plasma is routinely conducted to remove contaminants and promote wetting.^13–15^ However, these preparatory steps do not guarantee complete pore wetting after immersion in the electrolyte. During experiments, improper wetting may persist, and corrective measures, including the application of pressure or high-voltage pulses, are often required to establish stable ionic conduction and suppress noise.^16,6^ In order to plan such interventions, it is necessary to reliably detect the state of the pore, wetted or unwetted.

Conventional stability assessments rely primarily on subjective visual inspection of current–time traces to gauge baseline drift,^12^ a practice impractical for multiplexed monitoring involving numerous nanopores simultaneously. While transmission electron microscopy (TEM) imaging can provide some insight,^17^ it remains impractical and unreliable due to potential changes in pore conditions after imaging. Alternative approaches reported in the literature involve monitoring variations in resistance, hysteresis, and ionic current rectification (ICR) obtained from IV characterization.^16^ These metrics help assess pore quality before biomolecular measurements, but they are not inherently designed to evaluate wettedness during translocation experiments without an IV sweep.

Waugh et al. attempted to establish objective metrics, introducing the *L*-value to determine pore usability.^7^ *L*-value measures the 1/*f* noise of the pore up to 100 Hz, and if the obtained value exceeds the user-defined threshold, the pore is considered unusable. However, the threshold is specific to pore size and use case. Therefore, our work aims to identify robust, quantitative indicators of nanopore wettedness that remain valid across different applied voltages, pore dimensions, and short time segments suitable for real-time monitoring.

To achieve this objective, we present a quantitative framework for assessing nanopore wettedness, a key indicator of stability, using noise power spectral density (PSD) analysis. We detail PSD fitting methodologies, identify the optimal noise model using root-mean-square error (RMSE), and introduce various weighted-fit strategies. From the optimized PSD fits, we extract noise coefficients to predict wetting-related nanopore stability using a straightforward classification method.

While prior work has mainly focused on the link between 1/*f* noise and wetting, here we show that both the low-frequency (1/*f*) and the white noise components carry diagnostic information about pore health. In particular, we find that the white-noise coefficient *b*, the 1/*f* coefficient *a*_2_, and *L*-value together identify insufficiently wetted or unstable pores more reliably than 1/*f* noise alone.

Furthermore, the resulting classifier maintains high accuracy across different voltages and pore diameters, confirming that the selected features are robust under different conditions. We also examine the behavior of these features on short time segments to mimic real-time monitoring. By applying the trained classifier to each segment, we demonstrate that PSD-derived parameters can support both initial pore screening and ongoing pore-state assessment. These results indicate a path toward automated, real-time monitoring of nanopore health in multiplexed, high-throughput systems.

## 2 Noise in Nanopores

In SSNs, the noise components are broadly divided into four different classes.^18–30^ Each class exhibits colored noise: pink and/or brown (low-frequency 1/*f* noise), white (thermal and shot noise, dominant at intermediate frequencies), and blue and violet (typically associated with dielectric and capacitive noise, which dominate at higher frequencies), as shown in Figure 1. The noise characteristics (magnitude) observed in solid-state nanopores have been shown to depend on experimental conditions such as electrolyte composition, salt gradients, pore dimension, as reported in prior studies.^20,31,32^

**Figure 1:**
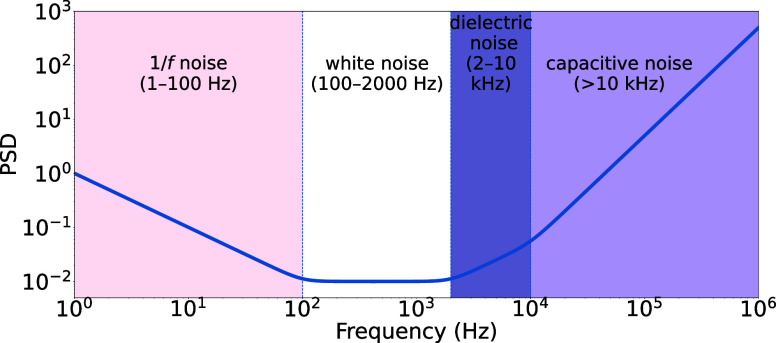
Noise PSD in log–log scale, schematically indicating the dominant noise regions across frequency ranges: 1/*f* (low-frequency), white, dielectric, and capacitive noise (adapted from ref 20, Copyright 2020 American Chemical Society). Note: the exact definition of the start point of the transition remains challenging, as the point where a particular noise component starts to dominate varies across experiments.^31,10^

### 2.1 Low-Frequency Noise

The 1/*f* noise originates from various sources, including nanobubbles, surface hydrophilicity (incomplete wetting), fluctuations in ion mobility, contamination during fabrication, and mechanical vibrations.^10^ 1/*f* noise is studied in the range up to 100 Hz. The noise PSD in the 1/*f* dominant region is represented by *S*_*I*,1/*f*_(*f*) and defined as follows:^31^

$$\frac{S_{I,1/f}(f)}{I^{2}}=\frac{a}{f}=\frac{\alpha }{N_{C}f}$$ 1

where *a* is the noise power, α is Hooge’s parameter, and *N*_C_ is the number of charge carriers.

### 2.2 White Noise

It includes thermal and shot noise and typically exhibits a flat PSD between 100 and 2000 Hz. However, in some pores, the dominant white-noise region may be displaced by 1/*f* or dielectric contributions. The PSD for white noise, *S*_*I*,white_ is defined as^20,10^

$$S_{I,\mathrm{white}}=\frac{4kT}{R}+2qI$$ 2

where $\frac{4kT}{R}$ is contribution from thermal noise and 2*qI* is from shot noise; here, *k* is the Boltzmann constant, *T* is temperature, *R* is resistance, *q* is the charge of a single carrier and *I* is ionic current.

### 2.3 Dielectric Noise

It is a high-frequency noise mainly due to the dielectric noise related to (pore membrane) capacitance. The PSD for dielectric noise, *S*_*I*,dielectric_(*f*), is defined as^20,10^

$$S_{I,\mathrm{dielectric}}(f)=8\pi kTDC_{\mathrm{chip}}f$$ 3

where *D* is dielectric loss and *C*_chip_, often termed parasitic capacitance, is the capacitance of the nanopore chip.

### 2.4 Capacitive Noise

Capacitive noise, also called amplifier or voltage noise, occurs in high-bandwidth systems (>10 kHz) and originates from both the nanopore chip capacitance and thermal voltage noise from the amplifier. The PSD for capacitive noise, *S*_*I*,capacitive_(*f*), is defined as^20,10^

$$S_{I,\mathrm{capacitive}}(f)=4\pi^{2}C_{\mathrm{tot}}^{2}v_{n}f^{2}$$ 4

where *C*_tot_ is the total capacitance (membrane + amplifier + others) and *v*_n_ is the input voltage noise from the amplifier.

The total noise PSD, *S*_*I*_(*f*), can generically be represented as the sum of all component PSDs:^20^

$$S_{I}(f)=\frac{a}{f}+b+cf+df^{2}$$ 5

where *f* is the frequency, and *a*, *b*, *c*, and *d* are the coefficients/strength of 1/*f*, white, dielectric, and capacitive noise, respectively. Adding the 1/*f* ^2^ component as in ref 33, (eq 5) becomes

$$S_{I}(f)=\frac{a_{1}}{f^{2}}+\frac{a_{2}}{f}+b+cf+df^{2}$$ 6

Another variant of the PSD model is presented in ref 34 as

$$S_{I}(f)=\frac{a}{f^{\beta }}+b+cf+df^{2}$$ 7

where, 0 ≤ β ≤ 2.

Combining eqs 6 and 7, we obtain a model:

$$S_{I}(f)=\frac{a_{1}}{f^{\beta_{1}}}+\frac{a_{2}}{f^{\beta_{2}}}+b+cf+df^{2}$$ 8

with 0 ≤ β_1_, β_2_ ≤ 2. This more flexible formulation is tested here as a possible extension for modeling multislope low-frequency noise, particularly when a single 1/*f* ^β^ component does not adequately describe the data. This approach has not, to our knowledge, been validated in the literature and is included for completeness and to assess its potential utility.

## 3 PSD Model Fitting: Nonlinear Least-Squares

We apply nonlinear least-squares^35^ curve fitting to analyze different noise components through the noise PSD model. The aim is to fit the model as defined in eqs 5–8.

Given the measured data points {(*f*_*i*_, *S*_*i*_)}_*i*=1_^*n*^, where *n* denotes the total number of frequency points, *f*_*i*_ is the *i*-th frequency point and *S*_*i*_ the corresponding PSD value, the model parameters **p** = [*a*_1_, *a*_2_, *b*, *c*, *d*]^T^ are estimated by minimizing the sum of squared residuals:

p^=argminp∑i=1nri2 9

where the residuals are defined as

$$r_{i}=S_{i}-\left(\frac{a_{1}}{f_{i}^{2}}+\frac{a_{2}}{f_{i}}+b+cf_{i}+df_{i}^{2}\right)$$ 10

Although negative parameter values may mathematically fit the curve, they lack physical meaning because noise contributions are strictly non-negative. Therefore, the parameters are constrained to [0, *∞*).

Weighted residuals are used to adjust the influence of frequency and PSD values:

$$r_{i,\mathrm{weighted}}=w_{i}r_{i}$$ 11

where *w*_*i*_ is a weight factor. In this study, four weighting schemes were tested (Table 1), selected to span a practical range of options. The choice of *w*_*i*_ directly influences the model fit quality and is thoroughly analyzed in Section 5.1.

**Table 1:** Details on Different Variants of Weights Used for Fitting the Noise PSD Model^a^

name	weight	description
NW	*w*_*i*_ = 1	no weight is used
EFS	$w_{i}=\frac{1}{f_{i}\cdot {\mathrm{S̃}}_{i}}$	equal weight given to frequency and PSD
HFLS	$w_{i}=\frac{1}{\sqrt{f_{i}}\cdot {\mathrm{S̃}}_{i}}$	higher weight on frequency, lower on PSD
LFHS	$w_{i}=\frac{1}{f_{i}\cdot \sqrt{{\mathrm{S̃}}_{i}}}$	lower weight on frequency, higher on PSD

a Here, *S̃* indicates the normalized PSD (See Section 4.6).

The model parameters are subsequently estimated by minimizing the sum of squared weighted residuals using nonlinear least-squares fitting:

p^=argminp∑i=1nri,weighted2 12

A robust initial guess is critical for the successful convergence of the fitting algorithm. Poor initial estimates may lead to slow convergence or suboptimal local minima.^36^ We obtained initial parameter estimates by individually fitting each noise component, using simple single-component models, over the frequency range where the component is most prominent (Table 2). For capacitive noise, it is reasonable to select initial ranges closer to the peaks in the high-frequency domain (e.g., 100,000–150,000 Hz for a 1 MHz bandwidth). The final parameters are derived from a model fit incorporating PSD values across the entire frequency range.

**Table 2:** Frequency Ranges and Single-Component Models Used for Obtaining Initial Parameter Estimates

parameter	frequency range	initial fit model
*a*_1_	0–15 Hz	*S*(*f*) = *a*_1_/*f* ^2^
*a*_2_	15–500 Hz	*S*(*f*) = *a*_2_/*f*
*b*	700–1000 Hz	*S* = *b*
*c*	7000–10,000 Hz	*S*(*f*) = *cf*
*d*	16,000–20,000 Hz	*S*(*f*) = *df* ^2^

These ranges indicate typical dominance regions, but they can vary significantly between experiments.^31,10^ Therefore, these ranges should be considered practical guidelines rather than fixed limits. In practice, these initial windows should be adjusted as needed. Automated methods may also assist in selecting these ranges. The initial frequency boundaries serve only as a reasonable starting point and do not constrain or fix the parameter values to the specified subranges.

Subsequently, these individual estimates combine to form the overall initial guess.

$$p_{0}=[a_{1},a_{2},b,c,d{]}^{T}$$ 13

Starting from **p**_0_, the parameter vector is iteratively updated until convergence:

$$p_{k+1}=p_{k}+\Delta p$$ 14

where Δ**p** is computed based on the local gradient and an approximation of the Hessian matrix.

Non-negativity constraints (**p** ≥ 0) are enforced using the trust-region reflective (TRF) algorithm,^37^ which adjusts proposed updates to ensure that

$$p_{j,k+1}=\max(0,p_{j,k}+\Delta p_{j})$$ 15

While the above formulation is presented for the five-component five-parameter (5C5P) model, the structure of the residuals *r*_*i*_ and the parameter vector **p** are modified as appropriate for the alternative noise PSD models examined in this study (see Table 3 and the analysis in Section 5.1). For each model, the overall fitting procedure and objective function remain the same, but the number and definition of parameters are adjusted to reflect the corresponding noise components (e.g., omitting or adding 1/*f* ^2^ or variable-exponent terms).

**Table 3:** Details on Different Variants of Noise PSD Model^a^

name	PSD model
4C4P	$S_{I}\left(f\right)=\frac{a}{f}+b+cf+df^{2}$ (eq 5)
4C5P	$S_{I}\left(f\right)=\frac{a}{f^{\beta }}+b+cf+df^{2}$ (eq 7)
5C5P	$S_{I}\left(f\right)=\frac{a_{1}}{f^{2}}+\frac{a_{2}}{f}+b+cf+df^{2}$ (eq 6)
5C7P	$S_{I}\left(f\right)=\frac{a_{1}}{f^{\beta_{1}}}+\frac{a_{2}}{f^{\beta_{2}}}+b+cf+df^{2}$ (eq 8)

a 4C4P = 4 components 4 parameters, 4C5P = 4 components 5 parameters, 5C5P = 5 components 5 parameters, and 5C7P = 5 components 7 parameters.

Similarly, the weighting schemes (Table 1) applied to the residuals are varied and systematically compared in later sections. The resulting set of fitted coefficients provides a quantitative summary of the noise contributions present in each open-pore current signal. These coefficients can serve as diagnostic features for subsequent data-driven analyses, particularly in the automated classification of pore wettedness and stability.

### 3.1 Use of Noise PSD Coefficients for Wettedness Classification

The fitted noise PSD coefficients were analyzed as features for classifying the nanopore state. In this study, we aim to distinguish two states: wetted (*y* = 0) and unwetted (*y* = 1).

For classification, we employ logistic regression,^38^ which is well-established for binary classification tasks. It is computationally efficient, performs reliably with moderate-sized datasets and low-dimensional feature sets, and has a convex objective that yields stable, reproducible training, making it suitable for practical deployment, including real-time analysis scenarios.^39^ Given the vector of PSD coefficients **p** = [*a*_1_, *a*_2_, *b*, *c*, *d*]^T^ (or a subset thereof, depending on the model), logistic regression learns an optimal set of weights **β** and intercept β_0_, such that the probability of a particular pore state (e.g., unwetted) is modeled as

$$P(y=1|p)=\frac{1}{1+\exp(-(\beta_{0}+\beta^{T}p))}$$ 16

where *y* is the binary pore state label. The model is trained to maximize the likelihood of the observed labels in the training set. Feature selection is described in Section 5.2. All classification models are implemented in Python using Scikit-learn with standard scaling.

## 4 Experiments

### 4.1 Data Acquisition

A total of 96 in-house fabricated SSNs (25 nm nominal diameter and 30 nm length) were tested. All pores were planar membrane nanopores, where the ionic sensing region is defined by an aperture in a thin SiN membrane on a Si-supported chip. The in-house pores were fabricated using deep-ultraviolet (DUV) lithography. Prior to measurements, chips were mounted in a flow cell and conditioned using standard cleaning and wetting procedures before filling with electrolyte. Experiments were conducted in 1 M KCl at pH 8.1. The solution conductivity ranged from 10.76 to 11.19 S m^–1^. Open-pore ionic current was recorded at a sampling rate of 200 kHz. No analytes were present during any measurement.

A total of 93 voltage-sweep experiments were performed, each involving one or more nanopores. Each pore participating in an experiment is counted as a pore-experiment combination, yielding 468 combinations in total. Individual pores appeared in 1–17 experiments (approximate mean of 5 experiments). Based on expert-defined criteria, 73 pores were consistently wetted, 21 consistently unwetted, and 2 exhibited mixed behavior across experiments. In total, 426 pore-experiment cases were wetted and 42 were unwetted.

For each pore-experiment combination, open-pore current segments of approximately four seconds were extracted at six applied voltages (50, 100, 150, 200, 250, and 300 mV). These segments inherited the label of their parent pore-experiment combination. The resulting dataset, TRAIN-MV, contains 5503 labeled segments (5011 wetted and 492 unwetted) and was used for mixed-voltage cross-validation (CV) and voltage generalization analysis. A subset of 812 segments selected at random from TRAIN-MV formed the model-comparison set for benchmarking the noise PSD models.

In addition, two independent test sets were collected under identical electrolyte and conductivity conditions. TEST-DIM contains 110 four-second segments from ten nanopores not included in TRAIN-MV: six in-house pores (30 nm length; diameters 20, 30, 50, and 200 nm), all unwetted, and four commercial planar nanopores (20 nm length; diameters 50 and 60 nm), all wetted. TEST-RT contains 770 one-second segments obtained by windowing continuous traces from 14 additional in-house 25 nm nanopores (30 nm length), including ten unwetted and four wetted pores, to emulate real-time monitoring.

Here, pore diameters are reported as nominal values (targeted design values for in-house pores and manufacturer specifications for commercial pores). For functional (wetted) pores, nominal diameters were corroborated from IV-sweep resistance using a standard conductance model;^40^ this is not applicable for unwetted pores.

All test-set nanopores were fully independent from the 96 pores used to construct TRAIN-MV. A summary of all datasets is provided in Table 4.

**Table 4:** Summary of the Datasets Used in This Study^a^

name	segments (W/U)	length	purpose
TRAIN-MV	5503 (5011/492)	4 s	CV, voltage generalization
TEST-DIM	110 (44/66)	4 s	dimension generalization
TEST-RT	770 (220/550)	1 s	real-time simulation

a All subsets have data with six applied voltages from 50 to 300 mV. W: wetted; U: unwetted.

### 4.2 PSD Estimation

The PSD of each open-pore current segment was estimated using Welch’s method^41^ with a Hann window, 50% overlap, and a frequency resolution of 5 Hz. These PSD estimates served as the input for noise model fitting and subsequent feature-based classification. As mentioned earlier, a subset of 812 PSD curves from TRAIN-MV was used for benchmarking the noise PSD estimation models.

### 4.3 Noise Model Fitting

Each PSD curve in the 812-segment subset was evaluated using the four noise PSD models listed in Table 3 combined with the four weighting schemes in Table 1, giving 16 model-weight combinations. By analyzing the fit quality (e.g., through the resulting RMSE), we discern which model and weighting strategy best captures the observed noise behavior in the pore signals. This approach ensures that each combination is directly comparable under the same experimental conditions.

### 4.4 Feature Selection Procedure

To determine which features are most informative for classifying wetted versus unwetted pores, an exhaustive subset evaluation was carried out. The candidate features were log *L*, *a*_1_, *a*_2_, *b*, *c*, *d*, *I*_RMS_, 5 kHz, ΔRMS, *V*. The coefficients *a*_1_, *a*_2_, *b*, *c*, and *d* were obtained by fitting the PSD using (eq 6). The additional features log *L* ^7^ and *I*_RMS_, 5 kHz^6^ were included to incorporate methods existing in the literature for assessing nanopore usability, and ΔRMS = |*I*_PEAK_ – *I*_RMS_, 5 kHz | was constructed as a simple deviation metric.

Comparing (eq 1) with the 1/*f* term in (eq 6) shows that, in the five-component model, *a*_2_ = *aI* ^2^. Even after normalizing the PSD as in (eq 17), the fitted 1/*f* coefficient still scales with *I* ^2^. To remove this dependence on the mean open-pore current *I*, we divide the fitted 1/*f* coefficient by *I* ^2^ and, for notational simplicity, use this current-normalized value as *a*_2_ in all classification experiments. All nonempty subsets of these features were then enumerated for exhaustive subset evaluation.

For each subset, a logistic regression model with fixed hyperparameters (Section 5.2) was trained using 10-fold stratified group cross-validation. “Stratified” ensures that the proportion of wetted and unwetted samples is preserved in each fold, and “group” ensures that all segments from the same pore-experiment combination remain in the same fold to prevent data leakage. The identification of the most informative feature set is reported in Section 5.2.

### 4.5 Pore Wettedness Classification

Logistic regression models were implemented with a tolerance of 1 × 10^–12^, regularization parameter *C* = 0.60, the newton-cholesky solver, and a maximum of 100,000 iterations. Feature sets were defined according to the feature-selection procedure described above.

Classifier performance was assessed using four evaluation protocols:

1. Mixed-voltage cross-validation: 10-fold stratified group cross-validation was applied to TRAIN-MV, with grouping by pore-experiment identity to prevent data leakage.
2. Generalization across applied voltages: models were trained on five voltages and tested on the remaining one, repeated for all six applied voltages.
3. Generalization across pore dimensions: the classifier trained on TRAIN-MV was applied without retraining to TEST-DIM (Table 4), which contains pores with dimensions not represented in the training set.
4. Feasibility of real-time pore health monitoring: the same classifier was evaluated on TEST-RT (Table 4), comprising one-second windows extracted from continuous recordings to mimic real-time monitoring.

Performance metrics are defined in Section 4.6 and Section S4 of the Supporting Information.

### 4.6 Evaluation Metrics

Model-fit quality was measured using RMSE of each PSD fit. PSDs were normalized as

$${\mathrm{S̃}}_{i}=\frac{S_{i}}{\sum_{j=1}^{n}S_{j}}$$ 17

ensuring comparability across segments and eliminating the need for additional RMSE normalization.

Classifier performance was quantified using standard metrics including accuracy, sensitivity (recall), specificity, precision, and F1-score (definitions provided in Section S4 of the Supporting Information).

## 5 Results and Discussion

### 5.1 PSD Model

Figure 2 presents the resulting RMSE distributions for all 16 model and weighting scheme pairs. Notably, although both model complexity and weighting shape the RMSE, the influence of the weighting scheme on fit quality becomes significant only when the model includes the core noise components, an effect we analyze in this section. Here, model complexity refers to the number of included noise components and associated fitting parameters, which increase progressively from 4C4P to 5C7P.

**Figure 2:**
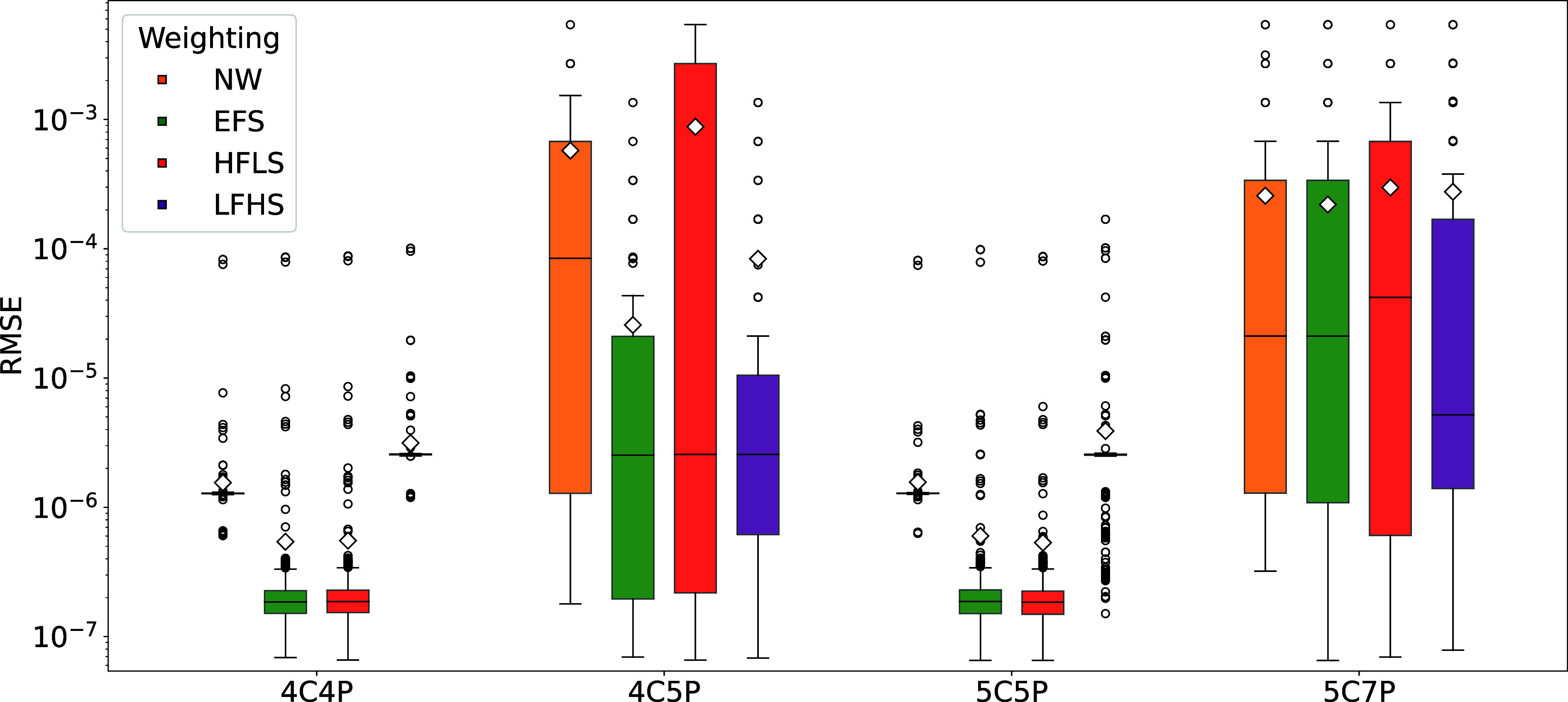
RMSE distributions (log scale) for all model and weighting scheme pairs. Each box shows the interquartile range (IQR), the horizontal line marks the median, the whiskers span the 5th−95th percentiles, and points beyond these limits are plotted as outliers. The diamonds denote the mean RMSE (numerical values reported in Table 5). The extremely tight interquartile ranges for 4C4P and 5C5P under NW and LFHS reflect consistent but systematically biased misfits, not superior accuracy. In contrast, 4C4P + EFS, 4C4P + HFLS, 5C5P + EFS, and 5C5P + HFLS exhibit both low median RMSE and compact distributions, identifying them as the most reliable combinations.

Stable fitting requires that the PSD model include each distinct noise component exactly once. The 4C4P model (with 1/*f*, white, dielectric, capacitive noise) performs well when a simple 1/*f* shoulder is sufficient, but underfits PSD that contain a clear 1/*f* ^2^ contribution. Adding the 1/*f* ^2^ term yields the 5C5P model, which assigns a unique parameter to each noise component and therefore achieves low, tightly clustered RMSE values. In 4C5P, the two low-frequency terms are replaced by a single variable-exponent term, so the optimizer must trade off between adjusting the exponent and its amplitude; the parameters become nonunique, and the RMSE distribution broadens. Extending the model to 5C7P introduces a second free exponent, allowing many amplitude-exponent pairs to reproduce nearly the same low-frequency curvature. The optimizer drifts among equivalent solutions, and both the median and the spread of the RMSE rise, which is a clear sign of nonidentifiability and overfitting due to redundant degrees of freedom rather than a lack of information in the PSD that could be remedied by additional data.

Once all core noise components are present in the model, the weights determine which frequency regions exert the most significant influence on the fit. With no weighting (NW), the high-frequency region contains far more data samples than the low-frequency region. As a result, the optimizer places most emphasis on reducing error at high frequencies. Because the PSD spans several orders of magnitude in frequency, this imbalance prevents the fit from matching any region well: the low-frequency 1/*f* and 1/*f* ^2^ shoulder, the white-noise floor, and the high-frequency rise are all poorly reproduced.

EFS weighting partially corrects this imbalance because low-frequency points with moderate amplitude receive comparatively higher weight. However, EFS assigns extremely small weights to data points where both frequency and PSD are large. In real PSDs, the dielectric and capacitive region contains both high frequency and high amplitude; under EFS, these data points contribute almost nothing to the objective, so the high-frequency rise becomes too flat. LFHS weighting reduces this suppression by penalizing large amplitudes less severely than EFS. This preserves the influence of large PSD values at low frequencies and improves the 1/*f* shoulder. However, the 1/*f*_*i*_ factor still dominates the weight, so high frequency data points continue to receive very little weight, and the capacitive rise remains underfitted.

By contrast, HFLS weighting reduces the rate at which weights decline more gradually with increasing frequency and moderates the influence of large amplitudes rather than eliminating it. This avoids the pitfalls of both EFS (which suppresses high frequency, high PSD data points too strongly) and LFHS (which still penalizes high frequencies excessively). Under HFLS, low-frequency data points retain enough influence to resolve the 1/*f* and 1/*f* ^2^ components, while high-frequency data points remain influential enough to constrain the dielectric and capacitive terms. All noise components, therefore, contribute meaningfully to the fit, which explains the consistently low RMSE of the 5C5P model with HFLS weighting in Figure 2 and the close agreement observed in the representative PSD fits shown in Figure 3.

**Figure 3:**
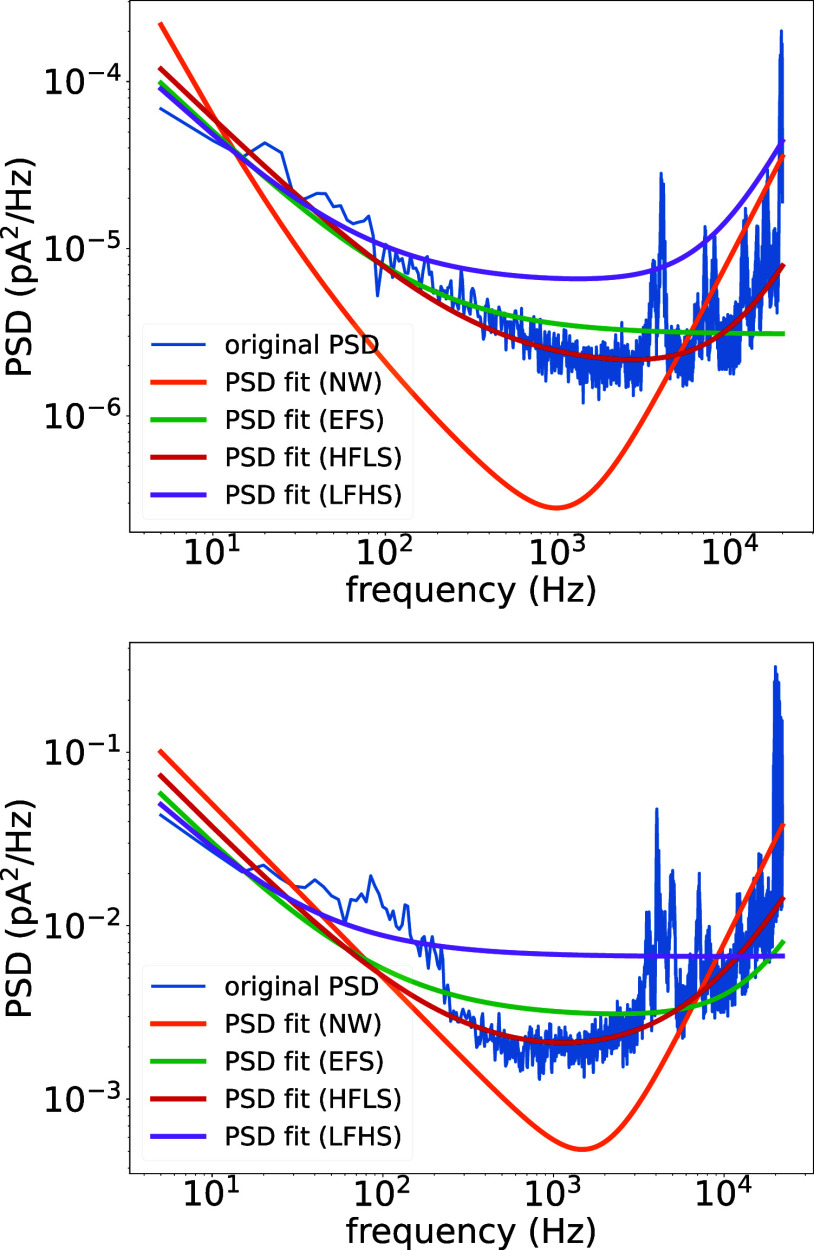
Representative PSD segments (blue) from wetted pores fitted with the 5C5P model under four weighting schemes. NW (orange) shows large deviations across the spectrum due to insufficient balancing of frequency contributions. EFS (green) improves the low-frequency region but suppresses the dielectric-capacitive rise. LFHS (purple) preserves more low-frequency structure yet still underestimates the high-frequency roll-up. HFLS (red) achieves the closest overall agreement, capturing both the 1/*f* region and the high-frequency rise, consistent with its lower RMSE in Figure 2. PSDs are shown on the raw (unnormalized) scale. For the top PSD segment, the corresponding unnormalized RMSE values are 1.41 × 10^–5^ pA^2^/Hz (HFLS) and 1.51 × 10^–5^ pA^2^/Hz (EFS); for the bottom segment, RMSE values are 0.0223 pA^2^/Hz (HFLS) and 0.0236 pA^2^/Hz (EFS).

Finally, these qualitative observations are confirmed quantitatively in Table 5, which presents the mean RMSE ± standard deviation. The 5C5P + HFLS combination achieves the lowest mean RMSE, closely followed by 4C4P + EFS and 4C4P + HFLS. This ranking confirms the box-plot trends and establishes 5C5P + HFLS as the most robust fitting strategy for nanopore noise PSD under the conditions tested. As long as the measured PSD exhibits the characteristic nanopore noise component structure illustrated in Figure 1, the HFLS weighting strategy is expected to remain applicable across different experimental conditions.

**Table 5:** Comparison of Four PSD Models (4C4P, 4C5P, 5C5P, and 5C7P) with Four Weighting Methods (NW, EFS, HFLS, and LFHS)^a^

weighting	4C4P	4C5P	5C5P	5C7P
NW	1.5524 × 10^–06^ ± 5.0000 × 10^–06^	5.7536 × 10^–04^ ± 9.5000 × 10^–04^	1.5662 × 10^–06^ ± 5.0000 × 10^–06^	2.5735 × 10^–04^ ± 5.8100 × 10^–04^
EFS	*5.4219 × 10^–07^ ± 5.0000 × 10^–06^*	2.5752 × 10^–05^ ± 7.3000 × 10^–05^	6.0118 × 10^–07^ ± 6.0000 × 10^–06^	2.2006 × 10^–04^ ± 5.4200 × 10^–04^
HFLS	5.5281 × 10^–07^ ± 5.0000 × 10^–06^	8.7902 × 10^–04^ ± 1.1740 × 10^–03^	**5.3341 × 10^–07^** ± **5.0000 × 10^–06^**	2.9789 × 10^–04^ ± 4.6600 × 10^–04^
LFHS	3.1479 × 10^–06^ ± 6.0000 × 10^–06^	8.3724 × 10^–05^ ± 2.7500 × 10^–04^	3.8957 × 10^–06^ ± 1.3000 × 10^–05^	2.7604 × 10^–04^ ± 7.8800 × 10^–04^

a Each cell shows (mean RMSE ± standard deviation). The best score is written in bold text, and the second-best score is italicized.

In summary, these results show that reliable PSD fitting requires a model that includes each of the relevant noise components and a weighting scheme that maintains balanced influence across all frequency regions. The 5C5P model combined with HFLS weighting satisfies both conditions, providing a stable, low-RMSE basis for extracting noise coefficients. We therefore adopt the 5C5P + HFLS combination in all subsequent experiments used for pore wettedness classification.

### 5.2 Pore Wettedness Prediction

An exhaustive feature search on the TRAIN-MV dataset identified the four-dimensional feature vector (log *L*, *a*_2_, *b*, *V*) as the most informative representation of pore wettedness. Table 6 reports only a curated subset of the evaluated models, namely the best-performing four-feature set, ablations with one feature removed, and baseline models using only PSD coefficients and voltage. Including any of the remaining candidates (*a*_1_, *c*, *d*, *I*_RMS_, 5 kHz, ΔRMS) did not improve the F1-score, and sometimes worsened it, showing that these additional features do not provide further discriminative information. Models based on a single low-frequency metric (log *L* only or *a*_2_ only, with or without *V*) also perform worse than the four-feature combination, confirming that any standalone feature is insufficient.

**Table 6:** Performance Metrics (Accuracy, Precision, Recall, Specificity, and F1-Score) for a Curated Subset of Feature Combinations Evaluated in the Exhaustive Search Reported as Percentages (%)^a^

feature set	accuracy	precision	recall	specificity	F1-score
log *L* + *a*_2_ + *b* + V	**99.71 [98.51–99.95]**	**98.68 [96.63–100.00]**	**100.00 [89.77–100.00]**	**99.90 [99.66–100.00]**	**98.00 [93.72–99.63]**
log *L* + *a*_2_ + *b*	99.33 [98.42–99.45]	97.17 [94.36–100.00]	96.21 [87.50–97.98]	99.71 [99.46–100.00]	95.36 [91.65–96.61]
*a*_1_ + *a*_2_ + *b* + *c* + *d* + *V*	97.77 [97.46–98.34]	100.00 [98.64–100.00]	77.84 [67.97–81.82]	100.00 [99.85–100.00]	87.20 [80.91–89.06]
log *L* + *b* + *V*	*99.53 [98.42–99.76]*	*97.21 [92.87–100.00]*	*100.00 [89.77–100.00]*	*99.71 [99.58–100.00]*	*95.62 [93.39–98.43]*
log *L* + *b*	99.33 [98.38–99.45]	96.90 [92.90–100.00]	96.21 [88.26–97.98]	99.60 [99.46–100.00]	95.20 [91.65–96.54]
log *L* + *a*_2_ + *V*	98.82 [98.38–99.40]	96.51 [91.77–99.55]	93.89 [85.38–100.00]	99.70 [99.30–99.95]	93.48 [90.89–95.18]
log *L* + *a*_2_	98.76 [98.36–99.38]	97.41 [91.59–100.00]	93.89 [85.38–100.00]	99.80 [99.23–100.00]	93.48 [89.41–95.51]
log *L* + *V*	98.59 [98.36–99.52]	94.63 [91.13–96.39]	95.41 [85.38–100.00]	99.49 [99.25–99.75]	93.48 [88.99–96.41]
log *L* only	98.59 [98.31–99.38]	96.10 [90.77–100.00]	98.48 [85.38–100.00]	99.61 [99.23–100.00]	93.48 [88.45–95.49]
*a*_2_ only	96.91 [95.89–97.60]	100.00 [95.45–100.00]	58.64 [53.93–72.36]	100.00 [99.80–100.00]	73.92 [69.02–82.93]
*a*_2_ + *V*	96.82 [95.99–97.60]	100.00 [95.45–100.00]	59.39 [54.39–72.65]	100.00 [99.80–100.00]	74.52 [69.39–83.51]
*a*_2_ + *b*	97.00 [96.10–97.69]	100.00 [97.76–100.00]	66.82 [56.73–72.65]	100.00 [99.85–100.00]	80.10 [72.39–83.51]
*a*_2_ + *b* + *V*	97.46 [96.90–97.80]	100.00 [98.47–100.00]	74.39 [61.08–76.63]	100.00 [99.80–100.00]	83.95 [75.83–85.37]

a Each cell shows median [25th percentile–75th percentile]. The results from the overall highest performing model are in bold font, and the second highest model results are shown in italics.

Except for *V*, all selected features are derived from the PSD. log *L* and *a*_2_ are measures of low-frequency noise, and their values increase when a pore is unwetted.^7,16^ This raises the question of why two similar metrics, log *L* and *a*_2_, are used. The reason is that log *L* only integrates the PSD up to 100 Hz, providing a robust summary of excess low-frequency noise. However, when the 1/*f* component extends beyond this range the low-frequency measure can become inaccurate; this gap is compensated by *a*_2_, which is obtained from the full-range PSD fit and captures the overall strength of the 1/*f* contribution independent of a predefined fixed frequency range. Hence, these two features complement each other.

Although the 1/*f* relation has been studied before,^7^ the behavior of the white-noise floor has rarely been explored as a practical indicator of pore condition. In this work, we revisit that relation and show that merging the white-noise coefficient *b* with the established low-frequency metrics boosts class separability.

Unwetted pores exhibit higher resistance,^16^ which lowers both thermal and shot noise (as shown in eq 2); the resulting drop in *b* therefore provides a complementary indicator that works alongside the 1/*f*-based metrics log *L* and *a*_2_. Adding *b* sharpens separation because the three coefficients move in opposite directions when a pore gets wetted. log *L* and *a*_2_ decrease as the 1/*f* region contracts, whereas *b* rises. These counter-directional shifts are clear in the normalized PSD overlays (Figures S6 and S8). The same plots show a short 1/*f* region plus a broad, flat white-noise plateau for wetted pores, versus a long 1/*f* tail and a narrowed white-noise band for unwetted pores (Figures S1–S5 and S7).

Together, log *L*, *a*_2_, and *b* describe both the integrated low-frequency power and the shape and relative contribution of the 1/*f* and white noise transition, while *V* captures systematic voltage dependence. This four-feature set therefore provides a compact, physically interpretable basis for wettedness classification. In all subsequent experiments, the logistic-regression classifier is trained and evaluated exclusively on (log *L*, *a*_2_, *b*, *V*). Even though the high-frequency coefficients are not used as features, we still fit the PSD with all five components. Because the frequency boundaries at which each noise term becomes dominant are not known a priori, fitting the full model is a conservative and robust choice.

#### 5.2.1 Mixed-Voltage Cross-Validation

In mixed-voltage cross-validation on TRAIN-MV, a single classifier trained on the feature set (log *L*, *a*_2_, *b*, *V*) generalizes well across the six applied biases (50–300 mV), achieving a median F1-score of 98.0% (Table 6). Despite the roughly 10:1 prevalence of wetted segments in TRAIN-MV, the median recall for the unwetted class remains above 95%, indicating that the classifier fully leverages the information contained in the fewer unwetted segments and does not collapse to predicting only the majority class. Hence, it was deemed that no additional class-balancing techniques were required.

As illustrated in Figure S8, log *L*, *a*_2_, and *b* vary with applied voltage. The increase in median F1-score from 95.36 to 98% when *V* is added to the feature set (log *L*, *a*_2_, *b*) (Table 6) shows that including *V* allows the model to account for this voltage dependence explicitly and to use a single voltage-robust classifier instead of models specific to each applied voltage bias.

#### 5.2.2 Generalization across Applied Voltages

Table 7 shows that the classifier maintains high performance when tested at voltages that were not present during training. For held-out voltages from 150 to 250 mV, F1-score stays around 96.5%, while at 300 mV it decreases to 95.56%. At 50 and 100 mV the F1-scores are lower (91.43 and 93.10%). The voltage dependence of these scores follows the feature distributions in Figure S8. For log *L*, *a*_2_, and *b*, the separation between wetted and unwetted pores is smallest and changes most steeply between 50 and 100 mV, then increases slowly between 100 and 250 mV and shows a small deviation at 300 mV.

**Table 7:** Results (in %) from Leave-One-Voltage-Out Testing^a^

voltage	accuracy	precision	recall	specificity	F1-score
50	98.50	90.91	91.95	99.12	91.43
100	98.80	96.43	90	99.67	93.10
150	99.40	100	93.33	100	96.55
200	99.40	100	93.33	100	96.55
250	99.40	98.84	94.44	99.89	96.59
300	99.20	95.56	95.56	99.56	95.56

a Voltage is in mV.

As a result, interpolation is most accurate in the 150–250 mV range where the feature-voltage relationship is almost linear. At 50, 100, and 300 mV the stronger nonlinearity or distortion yields slightly lower F1-scores, but they remain above 90%, indicating that the classifier still generalizes reliably to unseen operating voltages. More broadly, these results show that voltage-agnostic performance is best when the PSD-derived features follow a smooth, systematic trend with applied voltage, so that both interpolation and extrapolation in voltage space remain reliable.

#### 5.2.3 Generalization across Pore Dimensions

On the independent TEST-DIM subset (Table 4), the classifier trained only on 25 nm pores in TRAIN-MV, but tested on pores with diameters of 20, 30, 50, 60, and 200 nm, still maintains high performance. It achieves 98.18% accuracy, 100.00% precision, 100.00% specificity, 96.97% recall, and a 98.46% F1-score. TEST-DIM contains six unwetted in-house pores and four wetted commercial pores with different pore diameters and membrane thicknesses. One might suspect that the classifier is distinguishing in-house from commercial devices rather than the wetting state, but commercial pores are absent from TRAIN-MV, so the model cannot have learned such a label and must instead respond to differences in the noise PSD.

The PSD normalization is central to this generalization. It suppresses scale changes due to pore dimensions and preserves the characteristic spectral shape of each wetting state. As shown in Figures S6 and S7, normalized PSDs from wetted pores form a family with a relatively short 1/*f* region that is mostly confined below about 100 Hz and a broad, flat white-noise plateau, whereas unwetted pores share an extended 1/*f* shoulder that often reaches beyond 1 kHz and a much narrower white-noise region. The PSD-derived features *a*_2_ and *b* are extracted from these normalized PSD and capture the relative strengths of the 1/*f* and white-noise components, so a classifier trained on 25 nm pores can use the same shape and relative-contribution differences to identify the wetting state in solid-state nanopores with substantially different diameters and membrane thicknesses.

#### 5.2.4 Feasibility of Real-Time Pore Health Monitoring

On TEST-RT, the classifier achieves a mean accuracy of 96.75%, precision of 100.00%, specificity of 100.00%, recall of 95.45%, and an F1-score of 97.67% at the one-second segment level. These results indicate that the (log *L*, *a*_2_, *b*, *V*) feature set and logistic-regression model remain stable on one-second windows and can support real-time pipelines in which PSDs are recomputed at user-defined intervals to track pore state. In practice, the update interval can be matched to the experiment: for high-throughput measurements with thousands of translocations per minute, monitoring on the order of seconds to 1 min would allow rapid detection and correction of pore instability, whereas for slower measurements longer intervals of several minutes should be sufficient.

The proposed framework is designed to be compatible with real-time pore monitoring workflows. Model training is performed offline once, while prospective online operation would consist of repeated PSD computation, fitting of the noise PSD model to extract noise coefficients, and application of a logistic-regression classifier. Timing benchmarks performed on standard laptop hardware (Intel Core i7-1185G7 CPU, 16 GB RAM, Linux, Python 3.9) show that processing a one-second data segment requires under 200 ms, which is well below the duration of the data segment itself. As this benchmark was obtained using a Python implementation, reduced execution time can be achieved with optimized implementations in C or C++. This indicates that the computational requirements are feasible for continuous pore monitoring use cases.

The current feature set primarily captures two degradation pathways: (i) incomplete wetting or partial blockage, and (ii) unstable baseline conditions associated with drift, both reflected in elevated low-frequency noise. A third pathway, gradual enlargement of the pore due to electrochemical erosion during prolonged operation, is not directly monitored here. Because changes in pore diameter can be inferred from the open-pore current or resistance using standard conductance models for solid-state nanopores,^7^ future extensions could incorporate dynamically estimated resistance or conductance as an additional feature to detect pore enlargement alongside instability and wetting failure.

Across mixed-voltage cross-validation, generalization across applied voltages and pore dimensions, and real-time feasibility tests, the classifier based on (log *L*, *a*_2_, *b*, *V*) consistently distinguishes wetted from unwetted pores with high accuracy and robustness. Its reliance on physically interpretable PSD-derived parameters together with the applied voltage makes the approach experimentally practical and enables informed, automated assessment of nanopore state for pore-selection and real-time pore health monitoring in sensing applications.

## 6 Conclusions

This work presents a quantitative framework for assessing solid-state nanopore wettedness, an important indicator of operational stability, using power spectral density (PSD) analysis of the noise spectrum of the measured ionic current signal. By systematically comparing multiple PSD models and weighting schemes, a five-component, five-parameter (5C5P) model with high-frequency, low-PSD (HFLS) weighting was identified as the most robust configuration for accurately capturing nanopore noise characteristics. The coefficients derived from this optimized fitting, particularly the low-frequency component (*a*_2_) and the white-noise component (*b*), serve as physically interpretable indicators of pore health.

Using the PSD-derived coefficients (*a*_2_ and *b*) together with log *L* and the applied voltage (*V*) as features, we trained a logistic regression classifier that reliably distinguished wetted and unwetted pores with high accuracy across diverse conditions. The model achieved robust generalization across mixed voltages, unseen applied voltages, and pores of different dimensions, maintaining F1-scores above 90%. Real-time deployment was not implemented in hardware during this study; however, segment-by-segment evaluation on short time windows demonstrated that the proposed framework can operate effectively in a streaming setting, supporting its suitability for integration into continuous monitoring workflows.

Overall, this study establishes that PSD-based noise coefficients provide a compact, physics-informed feature set for robust pore-state assessment. The method offers a path toward automated, real-time nanopore quality monitoring, enhancing the reliability of multiplexed, high-throughput solid-state nanopore sensing platforms.

## Supplementary Material



## Notes

Example PSD fits across wetted and unwetted pores, absolute and normalized PSD plots, distributions of PSD-derived parameters across applied voltages, and classification metrics (PDF)

## Notes

Nilesh Madhu currently holds the Chair of Signal Processing for Medical Applications at Helmut-Schmidt-Universität/Universität der Bundeswehr Hamburg, Holstenhofweg 85, 22043 Hamburg, Germany.
