# Supplementary material for: Power Spectral Density Analysis of Solid-State Nanopore Signals: Application to Stability Estimation
Source: ACS Omega. 2026 Apr 14;11(16):24377–86. doi: 10.1021/acsomega.6c00036 (PMC13129830; doi:10.1021/acsomega.6c00036)
Supplement: Supplementary file 1 [file ao6c00036_si_001.pdf]

Power spectral density analysis of solid-state nanopore signals: application to stability estimation

Pratima Upretee[1], Eric Beamish[2], Wouter Botermans[2], Matteo Pero Cartiglia[2], and Nilesh Madhu*[1]

[1]IDLab, Department of Electronics and Information Systems, Ghent University - imec, Technologiepark Zwijnaarde 122, Ghent, 9052 Belgium
[2]imec, Kapeldreef 75, Leuven, 3001 Belgium

{pratima.upretee, nilesh.madhu}@ugent.be, {eric.beamish, wouter.botermans, matteopero.cartiglia}@imec.be

*Nilesh Madhu currently holds the Chair of Signal Processing for Medical Applications at Helmut-Schmidt-Universität/Universität der Bundeswehr Hamburg, Holstenhofweg 85, 22043 Hamburg, Germany.

Supporting Information

Contents

Supporting Information **S1**

S1 Example PSD fits across wetted and unwetted pores **S2**
S1.1 Parameter definitions S2

S2 Absolute and normalized PSD plots **S8**

S3 Distributions of parameters across applied voltages **S10**

S4 Classification metrics **S11**

S1 Example PSD fits across wetted and unwetted pores

S1.1 Parameter definitions

Following Waugh *et al.* [1], the L-value is defined as

$$L = \sqrt{\frac{1}{I^2} \int_1^{100} S(f) \, \mathrm{d}f}, \tag{1}$$

where $S(f)$ is the current noise power spectral density (PSD) between 1 and 100 Hz and I is the mean open-pore current. In this work, we use $\log L$ as a feature that summarizes the low-frequency noise level of the pore.

The PSD fits shown in Figures S1-S5 are based on the five-component, five-parameter (5C5P) model

$$S_I(f) = \frac{a_1}{f^2} + \frac{a_2}{f} + b + cf + df^2, \tag{2}$$

where each coefficient corresponds to a distinct physical noise contribution:

- a_1: coefficient of the $1/f^2$ term
- a_2: coefficient of the $1/f$ term
- b: white-noise coefficient (thermal + shot noise floor)
- c: dielectric noise coefficient
- d: capacitive/amplifier noise coefficient.

Unless otherwise stated, a_2 denotes the normalized $1/f$ coefficient (i.e., divided by I^2), and the quantities a_2, b, and $\log L$ are the parameters reported in the supplementary figures. The HFLS weighting scheme gives frequency significant priority over the PSD values.

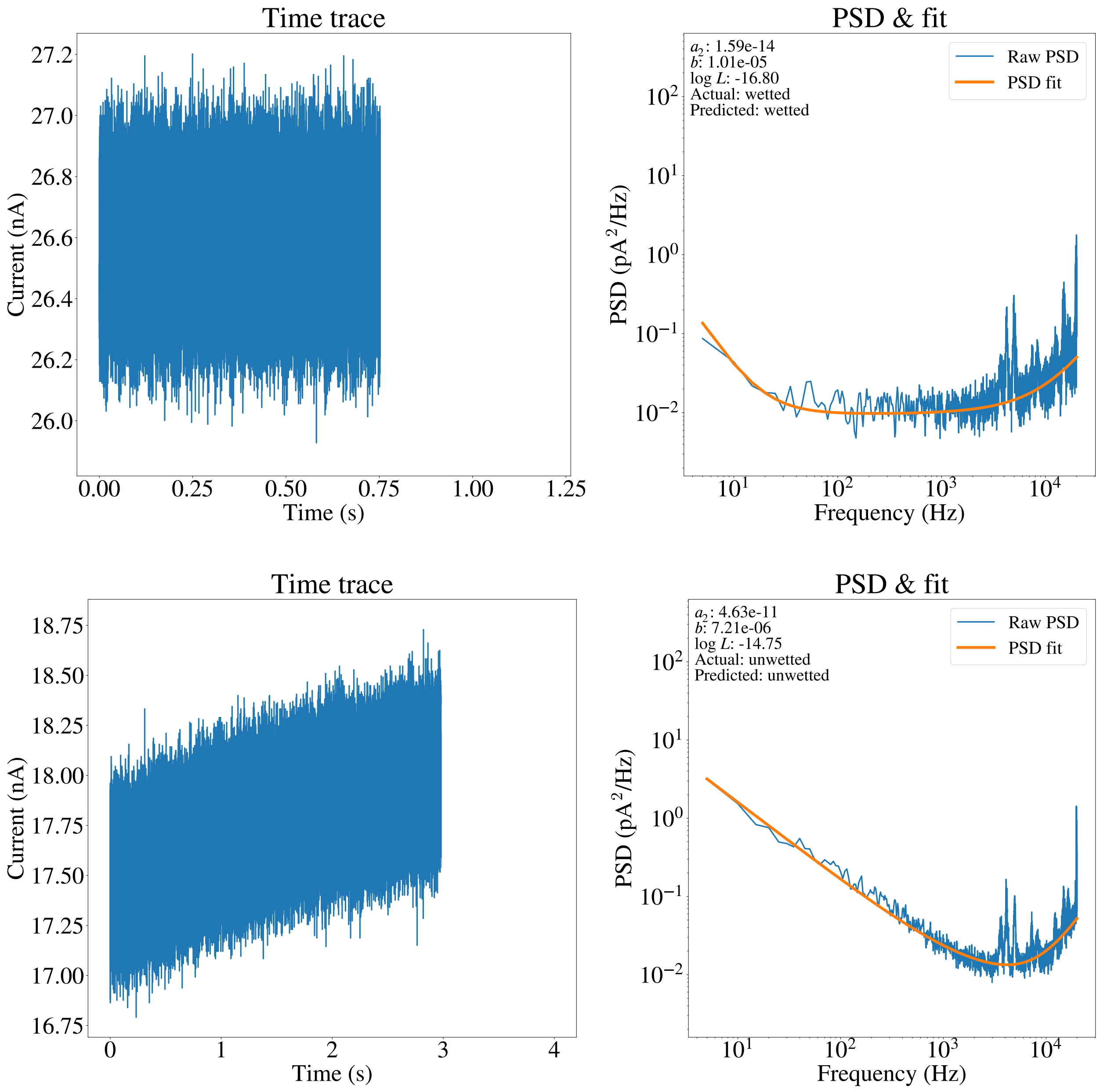

Figure S1: Representative PSD fits for a wetted pore (top) and an unwetted pore (bottom), example 1.

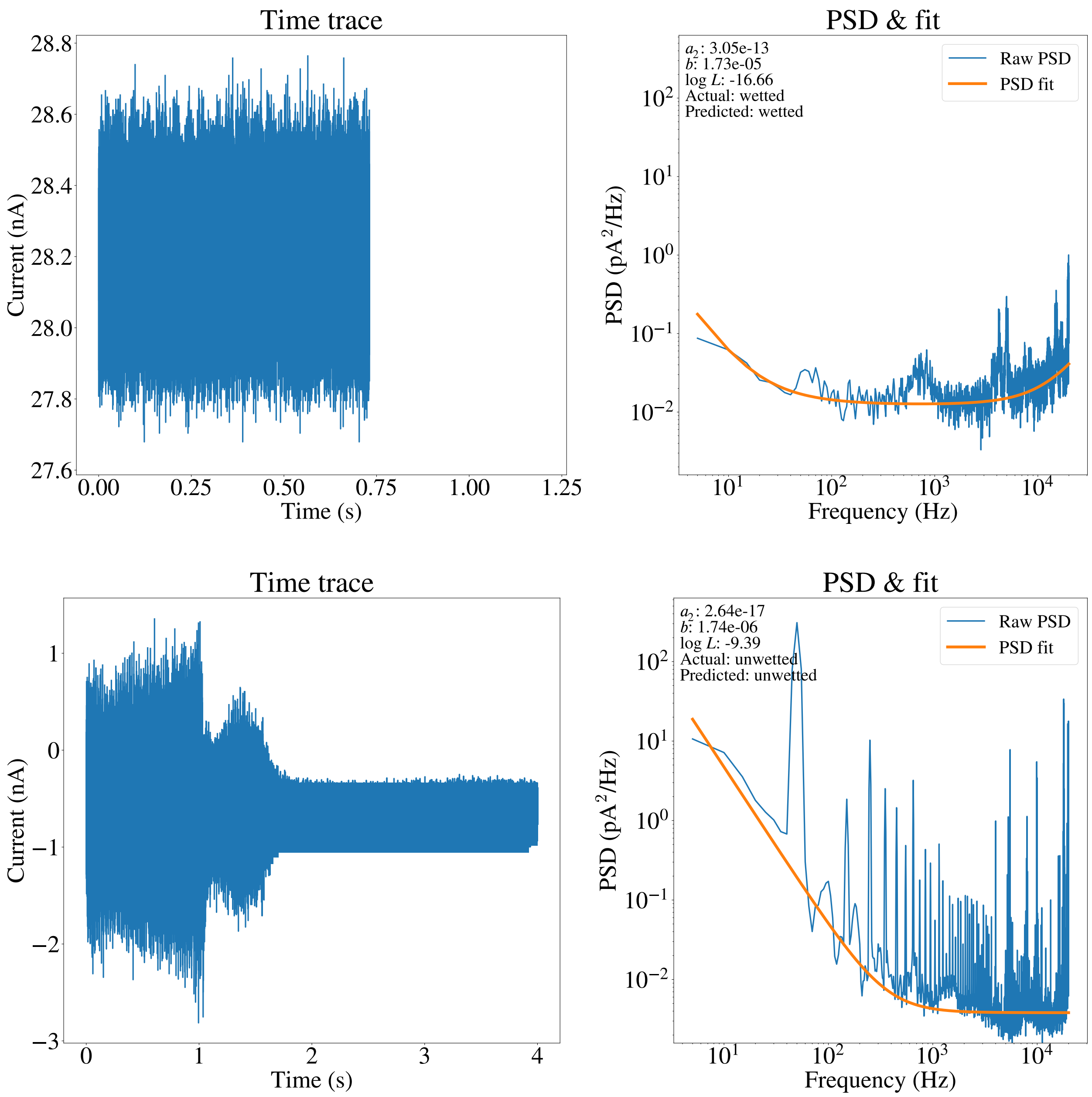

Figure S2: Representative PSD fits for a wetted pore (top) and an unwetted pore (bottom), example 2.

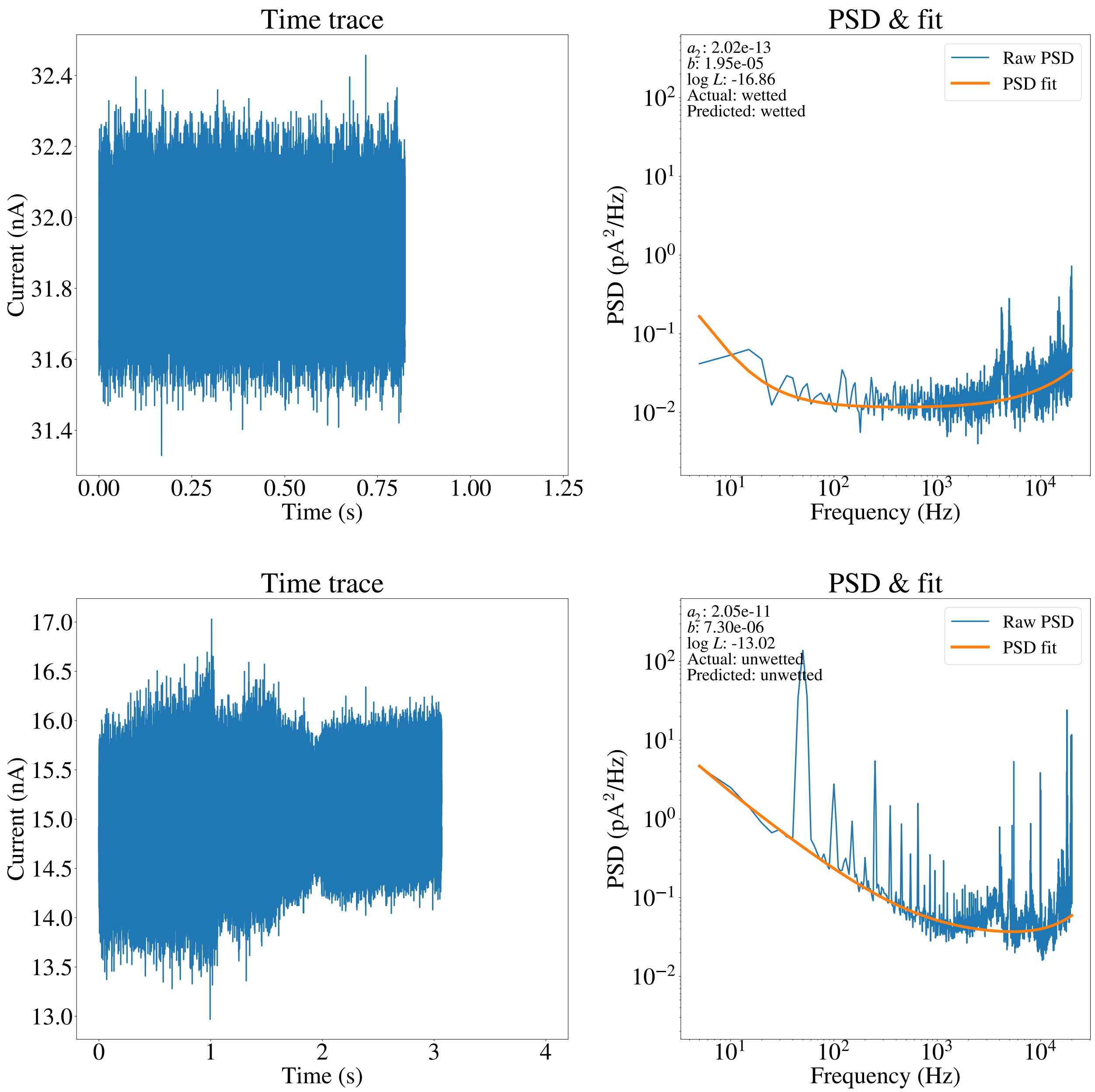

Figure S3: Representative PSD fits for a wetted pore (top) and an unwetted pore (bottom), example 3.

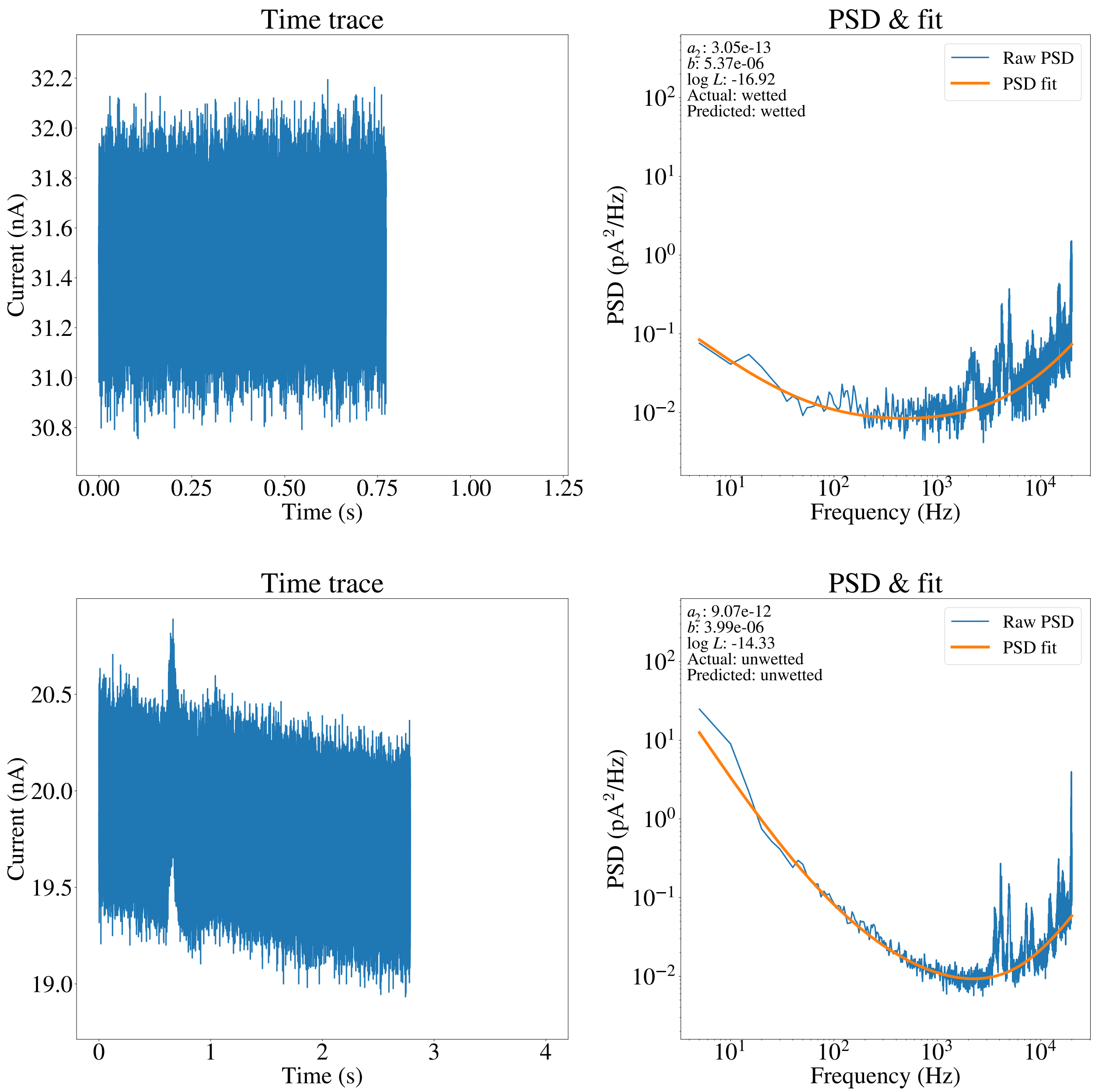

Figure S4: Representative PSD fits for a wetted pore (top) and an unwetted pore (bottom), example 4.

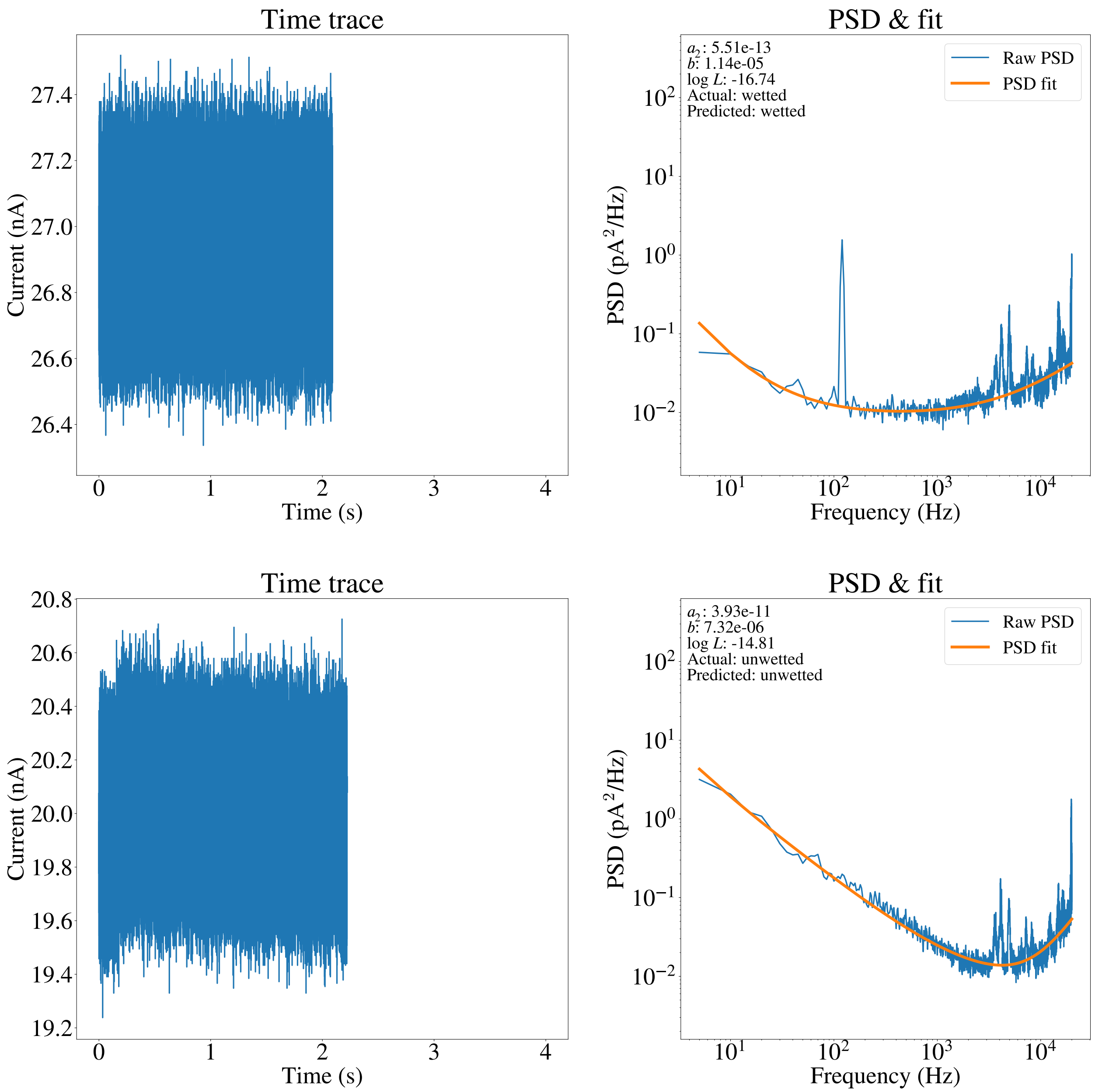

Figure S5: Representative PSD fits for a wetted pore (top) and an unwetted pore (bottom), example 5.

S2 Absolute and normalized PSD plots

This section compares the absolute noise PSDs with their normalized counterparts to illustrate how normalization emphasizes the relative contribution of different noise components.

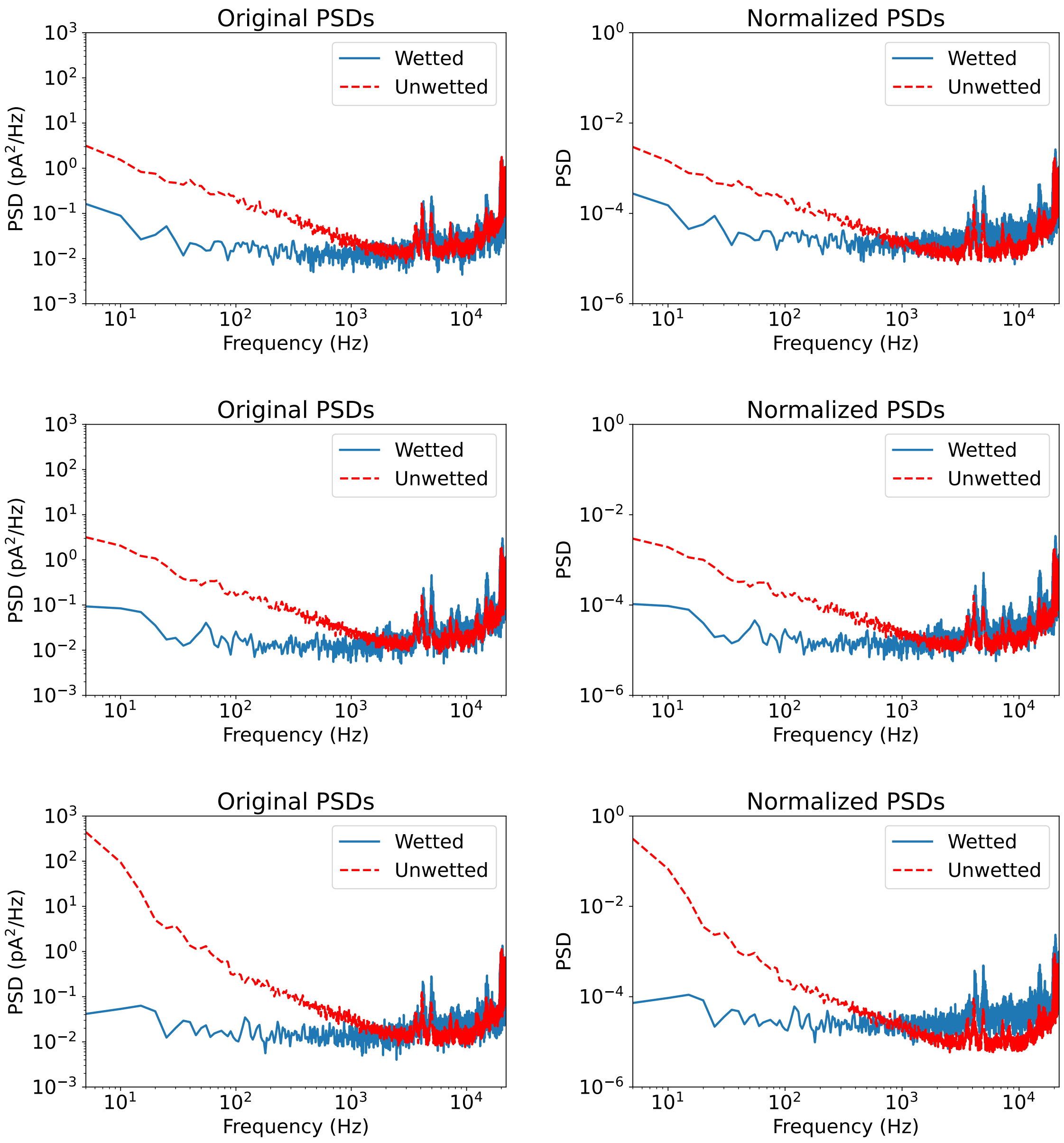

Figure S6: Noise PSDs for wetted and unwetted pores. Left: absolute PSDs; right: normalized PSDs. Normalization highlights shape-based differences in the PSD, particularly the reduced white-noise region for unwetted pores, which are less evident in the absolute PSDs.

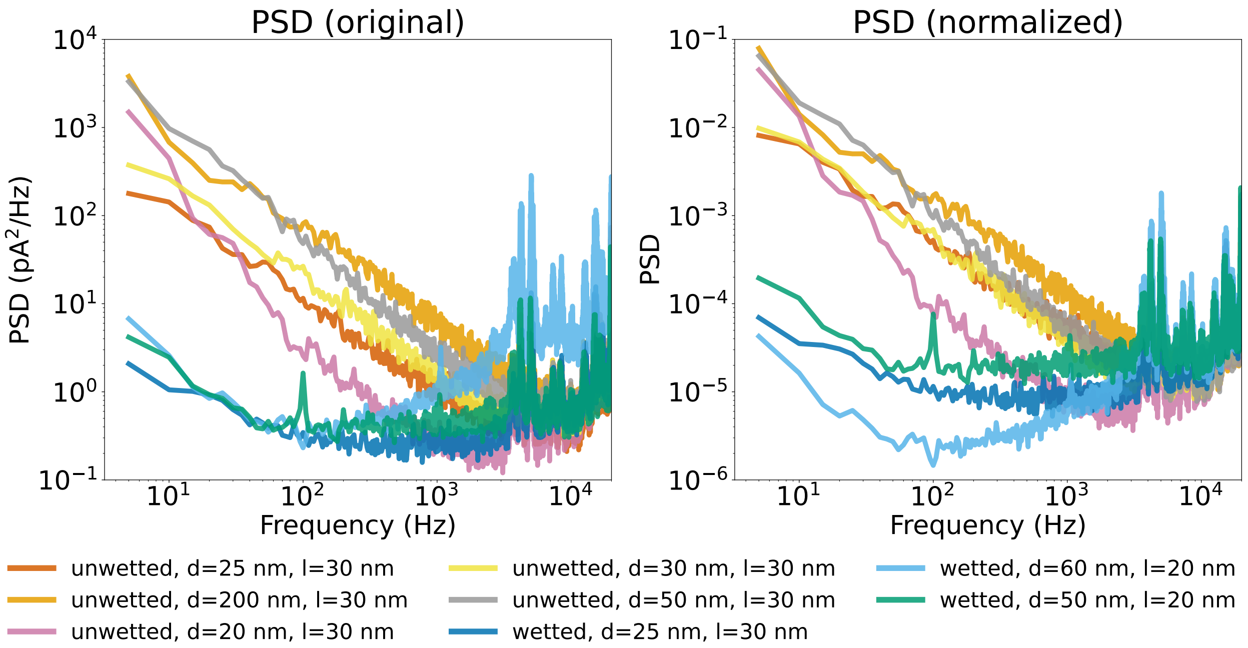

Figure S7: Noise PSDs for wetted and unwetted pores of different dimensions. Left: absolute PSDs; right: normalized PSDs. The distinct shape with steep $1/f$ slope and narrow white-noise region is observed for unwetted pores, while wetted pores have flatter $1/f$ slope and wide white-noise regions.

S3 Distributions of parameters across applied voltages

This section shows the distributions of the fitted PSD parameters (a_2, b, and $\log L$) across the applied voltage range 50–300 mV. Consistent with the trends discussed in the main text, all three features exhibit their smallest separation between wetted and unwetted pores at 50 mV. Between 50 and 100 mV the mean values of each feature diverge sharply, producing a markedly nonlinear increase in the ratio of wetted to unwetted feature magnitudes. Above 100 mV the voltage dependence becomes much gentler, with the feature trajectories changing more nearly linearly up to 300 mV. These patterns explain the performance at 50 mV in voltage generalization analysis test. When 50 mV is held out, the classifier must extrapolate across the steep 50–100 mV transition, whereas for all voltages above 100 mV the smoother trend preserves strong generalization (F1 > 96%).

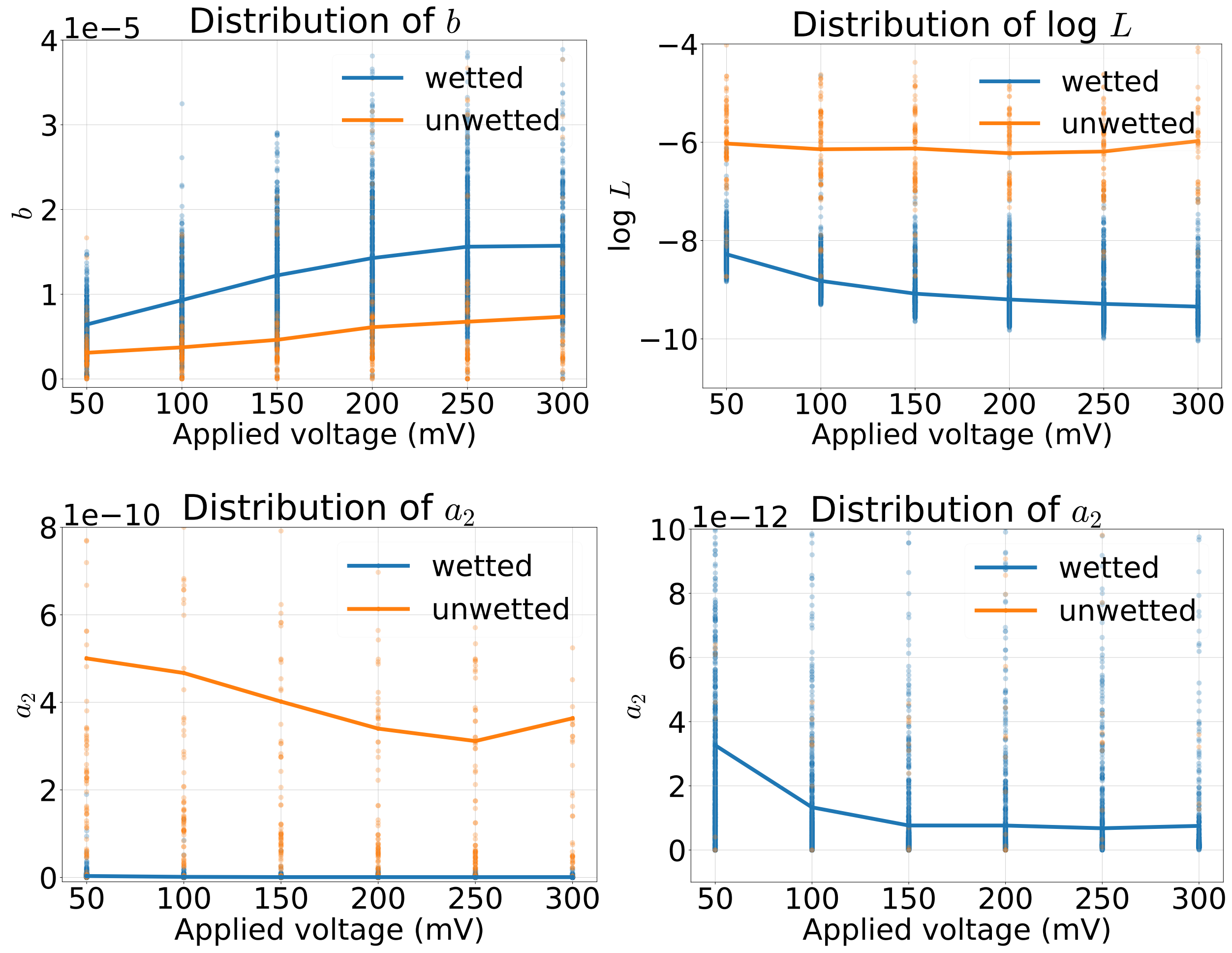

Figure S8: Distributions of PSD-derived features across the applied voltages 50–300 mV for wetted and unwetted pores. Top row: (left) b (white-noise coefficient) and (right) $\log L$. Bottom row: (left) normalized a_2 and (right) the same data on a zoomed axis. All three features show their smallest class separation at 50 mV. Between 50 and 100 mV the feature means diverge sharply, producing a nonlinear rise in the ratio of wetted to unwetted values, whereas above 100 mV the dependence becomes more gradual and nearly linear. These trends align with the generalization across applied voltages in the main text: classification at 50 mV is more difficult due to the steep 50–100 mV transition, while the smoother progression at higher voltages supports strong voltage-agnostic generalization.

S4 Classification metrics

Classifier performance was evaluated using five standard metrics: true positive rate (TPR), true negative rate (TNR), precision, F1-score, and accuracy. These metrics are defined as follows:

$$\text{TPR (recall or sensitivity)} = \frac{\text{TP}}{\text{TP} + \text{FN}}, \tag{3}$$

$$\text{TNR (specificity)} = \frac{\text{TN}}{\text{TN} + \text{FP}}, \tag{4}$$

$$\text{Precision} = \frac{\text{TP}}{\text{TP} + \text{FP}}, \tag{5}$$

$$\text{F1-score} = 2 \cdot \frac{\text{Precision} \cdot \text{Recall}}{\text{Precision} + \text{Recall}}. \tag{6}$$

$$\text{Accuracy} = \frac{\text{TP} + \text{TN}}{\text{TP} + \text{FP} + \text{TN} + \text{FN}}. \tag{7}$$

Here,
TP = unwetted pore is correctly classified as unwetted.
TN = wetted pore is correctly classified as wetted.
FP = wetted pore is falsely classified as unwetted.
FN = unwetted pore is falsely classified as wetted.

TPR quantifies the proportion of unwetted pores correctly identified, while TNR measures the proportion of correctly identified wetted pores. Precision measures the fraction of predicted unwetted pores that are actually unwetted. The F1-score reflects the harmonic mean of precision and recall; an F1-score of 100% indicates perfect precision and recall. Accuracy measures the proportion of all pores that are correctly classified.

References

[1] M. Waugh, K. Briggs, D. Gunn, M. Gibeault, S. King, Q. Ingram, A. M. Jimenez, S. Berryman, D. Lomovtsev, L. Andrzejewski, *et al.*, "Solid-state nanopore fabrication by automated controlled breakdown," *Nature Protocols*, vol. 15, no. 1, pp. 122–143, 2020.
